# Rotational Atherectomy for Ablation of a Retained Microcatheter Distal Tip in a Calcified Chronic Total Occlusion

**DOI:** 10.1016/j.jaccas.2026.108552

**Published:** 2026-07-15

**Authors:** Prathap Kumar, Youness Toukami, Manu Rajendran, Abhiram Katragadda, Thaha Mohamed Hussein, Roshan Ghimire, Blessvin Jino

**Affiliations:** a Meditrina Hospital, Kollam, Kerala, India; b CHU Ibn Rochd, Casablanca, Morocco

**Keywords:** burr entrapment, calcified coronary disease, chronic total occlusion, microcatheter tip entrapment, retained intravascular foreign body, rotational atherectomy

## Abstract

**Background:**

Retention of a microcatheter distal tip during chronic total occlusion (CTO) percutaneous coronary intervention (PCI) is rare and particularly challenging in heavily calcified, balloon-uncrossable lesions.

**Case Summary:**

A 66-year-old man with symptomatic chronic coronary syndrome underwent PCI for a severely calcified ostial left anterior descending artery CTO. After antegrade wire crossing, the lesion remained balloon-uncrossable. During balloon-assisted wire exchange, the Finecross distal radiopaque tip fractured and remained embedded within the CTO segment. Rotational atherectomy with a 1.5-mm burr resulted in fluoroscopic disappearance of the retained tip while preserving TIMI flow grade 3. Because lesion crossability remained limited, a 1.25-mm burr was used for further plaque modification, possibly because interaction with the retained tip material reduced the initial burr's cutting efficiency. PCI was then completed with stent implantation and an excellent final angiographic result.

**Discussion:**

This case highlights rotational atherectomy as a bailout option in calcified uncrossable CTOs, while acknowledging the uncertain downstream risk of fragmented microcatheter material.

**Take-Home Message:**

Controlled plaque modification may permit safe PCI completion when retrieval is impractical.

## Visual Summary

**Figure:**
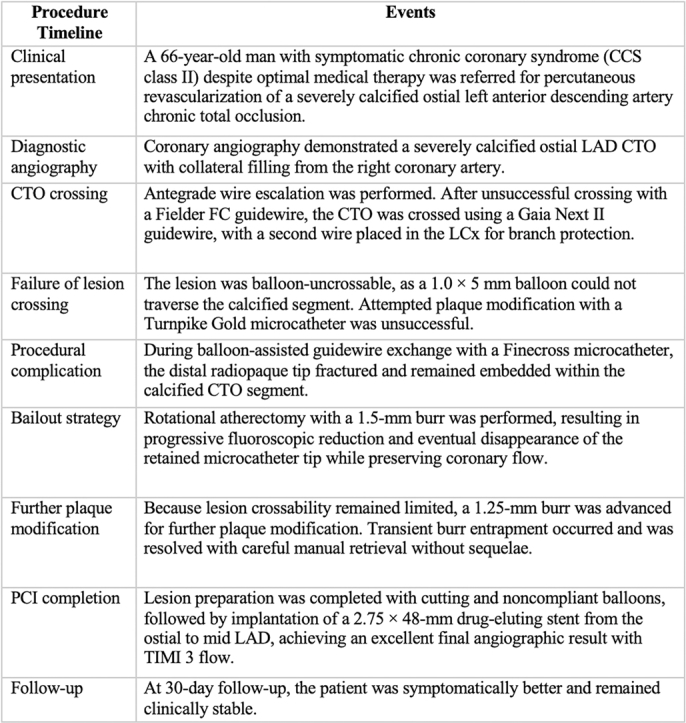

**Visual Summary:**
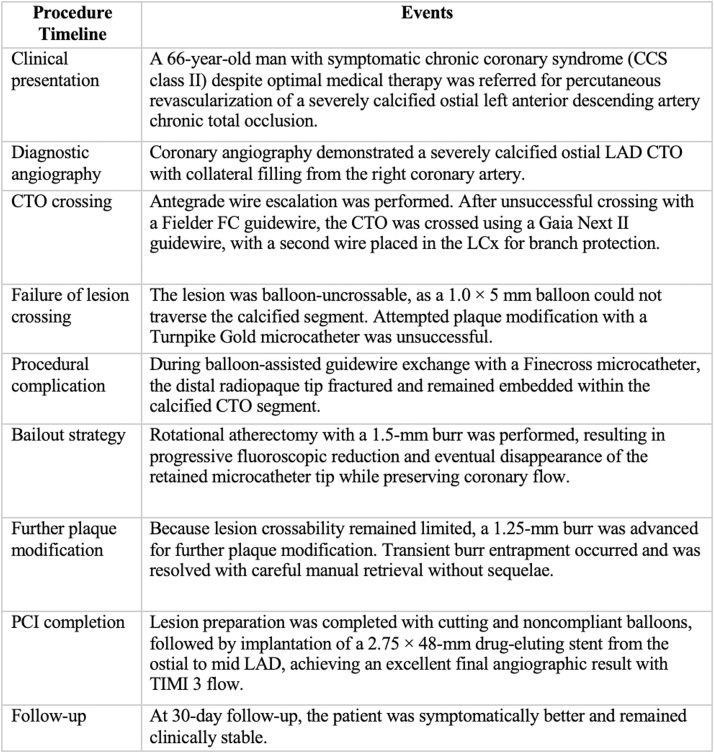
Timeline of the Case CTO = chronic total occlusion; LAD = left anterior descending artery; LCx = left circumflex artery; PCI = percutaneous coronary intervention.

## History of Presentation

A 66-year-old man with symptomatic chronic coronary syndrome (class II) despite optimal medical therapy was referred for percutaneous revascularization of an ostial left anterior descending artery (LAD) chronic total occlusion (CTO). He was hemodynamically stable, and physical examination was unremarkable.

## Past Medical History

Hypertension, diabetes mellitus, and a history of coronary artery disease on optimal medical treatment.

## Investigations

**Figure 1:**
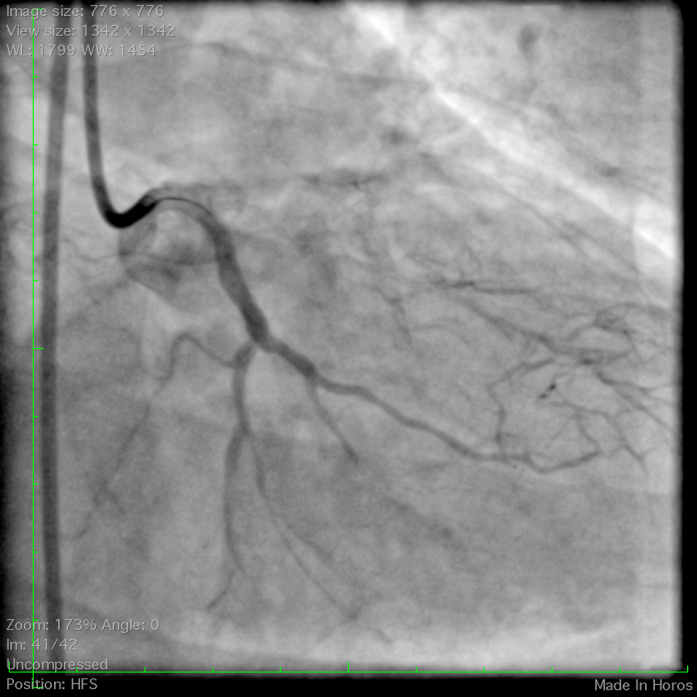
Diagnostic Angiography of the Left Coronary System Demonstrating a Severely Calcified Ostial Left Anterior Descending Artery Chronic Total Occlusion

**Figure 2:**
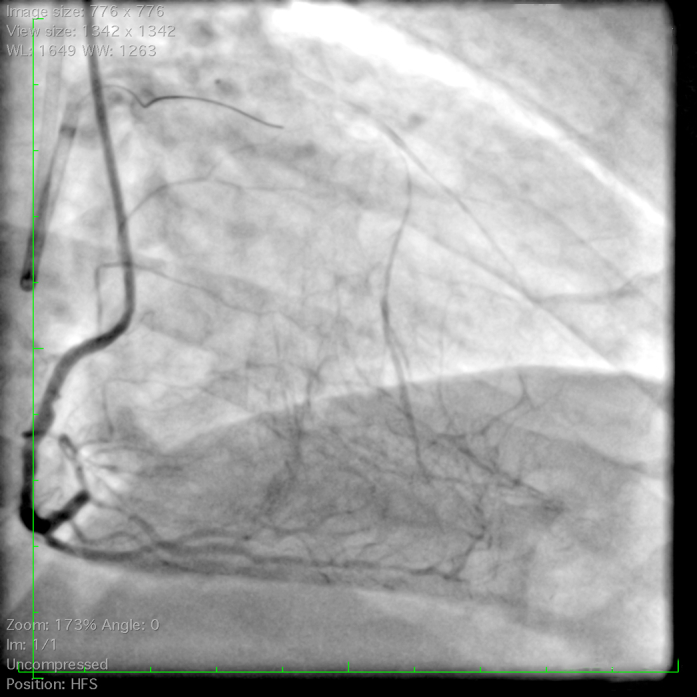
Diagnostic Angiography of the Right Coronary System Demonstrating Right-to-Left Collateral Filling to the Left Anterior Descending Artery Territory

Coronary angiography demonstrated a severely calcified ostial LAD CTO with collateral filling from the right coronary artery (Figures 1 and 2). Baseline electrocardiogram and transthoracic echocardiography were normal. Baseline renal function was normal, and routine blood tests were normal.

## Management

A 7-F EBU 3.0 guide catheter was engaged in the left main coronary artery. Antegrade wire escalation was performed with microcatheter support (Finecross MG; Terumo). After unsuccessful crossing with a Fielder FC guidewire, the CTO was successfully crossed using a Gaia Next II guidewire (Asahi Intecc). A second Fielder FC wire was positioned in the left circumflex artery for branch protection (Video 1).

Following successful wire crossing, the lesion was found to be balloon uncrossable, as a 1.0 × 5-mm balloon could not traverse the calcified segment. Initial plaque modification was attempted using a Turnpike Gold microcatheter; however, this strategy failed to cross the lesion.

Given the unavailability of excimer laser coronary atherectomy, rotational atherectomy was planned as the next step for calcium modification. A Finecross microcatheter was reintroduced over the Gaia Next II wire and forcefully advanced to wedge within the heavily calcified segment to facilitate exchange. During withdrawal using a balloon-trapping technique, resistance was encountered, and fluoroscopy revealed fracture of the distal tip, which remained embedded within the calcified CTO body, while the microcatheter shaft was successfully retrieved (Figure 2).

The guidewire was maintained in position to preserve distal access. A Turnpike Gold microcatheter was subsequently advanced to allow exchange for a RotaDrive wire. Considering that the fractured distal tip consisted of a relatively soft metallic component embedded within dense calcification, rotational atherectomy was undertaken with the intent to modify both the calcified plaque and the entrapped fragment.

**Figure 3:**
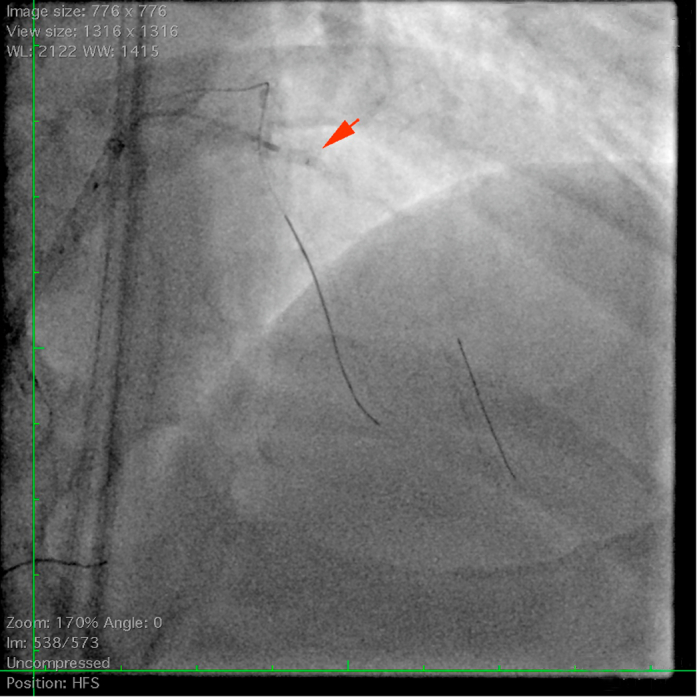
Fracture of the Finecross Microcatheter Distal Radiopaque Tip During Balloon-Assisted Guidewire Exchange, With Retention of the Tip Within the Calcified Chronic Total Occlusion Segment

A 1.5-mm burr was initially used at 180,000 rpm. Multiple short runs were performed (with cumulative ablation time of approximately 3.2 minutes). Progressive reduction and eventual fluoroscopic disappearance of the fractured microcatheter tip were observed, suggesting successful fragmentation/pulverization (Figure 3). However, despite apparent tip ablation, the calcific lesion remained balloon uncrossable, possibly due to insufficient plaque modification or altered burr dynamics (Video 2).

A smaller 1.25-mm burr was therefore selected and advanced at 200,000 rpm. After approximately 30 seconds of rotational run, the burr crossed the lesion but subsequently became transiently entrapped within the calcified segment. Careful manual traction allowed retrieval without sequelae.

**Figure 4:**
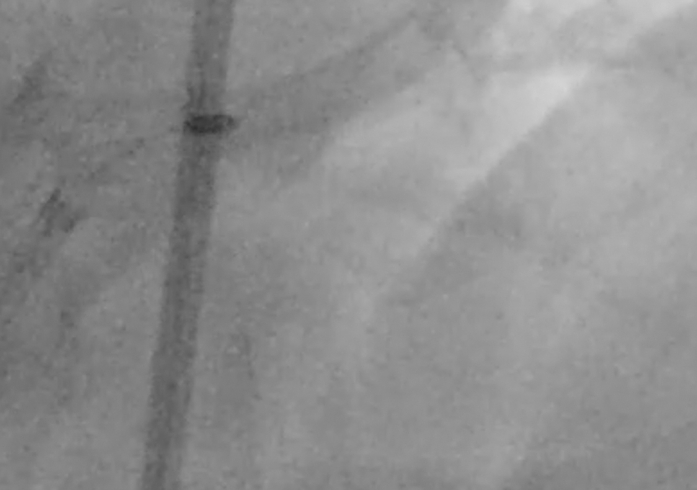
Fluoroscopic Disappearance of the Retained Microcatheter Distal Tip After Rotational Atherectomy Runs, Consistent With Ablation/Fragmentation of the Retained Marker

Lesion preparation was completed using a 2.0 × 12-mm cutting balloon, followed by a 2.5 × 15-mm noncompliant balloon. A 2.75 × 48-mm drug-eluting stent was deployed from the ostial to mid LAD, achieving an excellent angiographic result with the final TIMI flow grade 3 (Figure 4).

**Figure 5:**
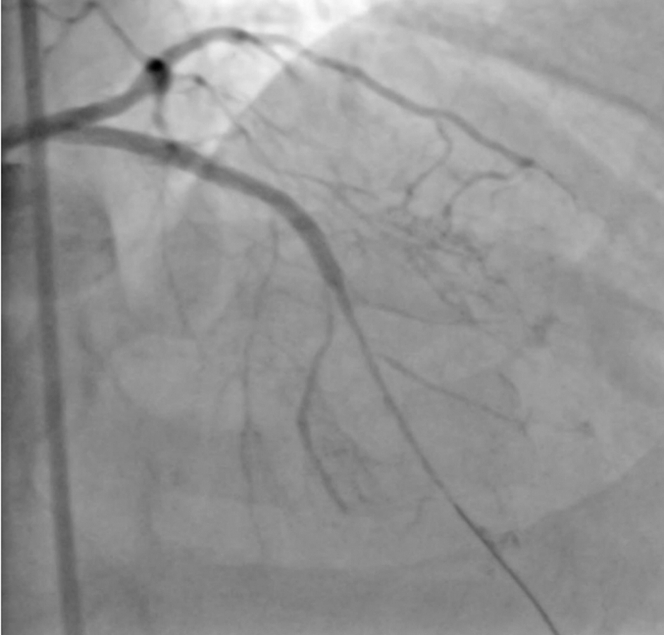
Final Angiographic Result After Calcium Modification and Drug-Eluting Stent Implantation, Showing Successful Revascularization of the Left Anterior Descending Artery with TIMI Flow Grade 3

**Figure 6:**
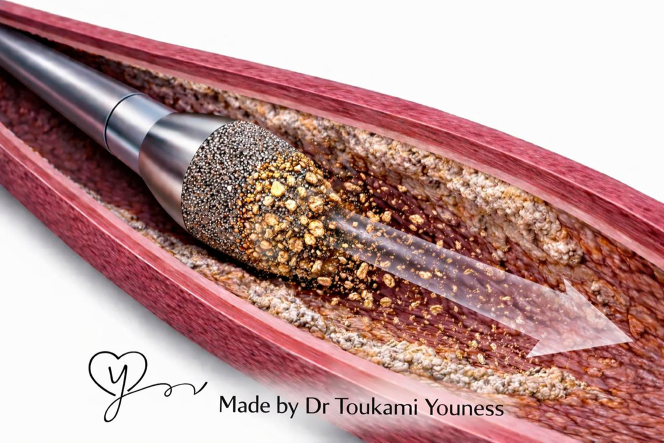
High-Resolution Illustration of Rotational Ablation of an Entrapped Microcatheter Tip Using Rotational Atherectomy

Activated clotting time was maintained between 250 and 300 seconds throughout the procedure.

## Outcome and Follow-Up

At 30 days, the patient was free from angina and reported mild exertional dyspnea (NYHA functional class II).

Electrocardiogram and transthoracic echocardiography were stable compared with baseline.

## Discussion

Device fracture with retention of a distal microcatheter tip within a calcified CTO creates a unique procedural dilemma. In this case, the retained Finecross distal marker was embedded within a balloon-uncrossable, heavily calcified ostial LAD CTO, preventing conventional lesion preparation and limiting retrieval options.

The critical issue was not simply foreign body retention, but obstruction within an already rigid and uncrossable segment. Because the lesion could not be crossed with a balloon, traditional extraction techniques were technically constrained. Manipulation also risked proximal vessel compromise in an ostial segment supplying a large myocardial territory. Prior reports have described percutaneous retrieval of retained intracoronary catheter fragments using techniques such as twisted wires and guide-extension support; however, such strategies are more applicable when the retained component is accessible and not deeply embedded within a calcified CTO body.^1^^,^^2^

The procedural strategy therefore shifted from retrieval to restoration of lumen patency. Rotational atherectomy was selected to modify the calcified plaque and regain lesion crossability. Although rotational atherectomy is primarily intended for plaque modification in heavily calcified coronary lesions, its controlled interaction with rigid obstructive material provided a potential bailout option in this setting.^3^^,^^4^ Following short, carefully monitored runs with a 1.5-mm burr, the radiopaque fragment was no longer visible fluoroscopically, and coronary flow remained preserved.

Although the retained tip was no longer visible fluoroscopically after 1.5-mm-burr runs, lesion crossability remained limited, suggesting that modification of the surrounding calcified plaque was still incomplete. In addition, the interaction between the burr and the retained microcatheter distal tip material may have reduced the cutting efficiency of the initial burr, although this remains speculative. Transient burr entrapment occurred during further plaque modification but was resolved with manual traction of the entire system without sequelae, allowing completion of percutaneous coronary intervention with stent implantation and the final TIMI flow grade 3 (Figure 5).

A similar but non-identical case of rotational atherectomy used to manage a fractured microcatheter tip has previously been reported by Alkhalil et al.^5^ However, in the present case, rotational atherectomy was undertaken with the specific aim of modifying both the surrounding calcified plaque and the retained distal tip material, with progressive fluoroscopic reduction and eventual disappearance of the fragment observed during the procedure.

A further consideration is the potential for distal embolization of fragmented microcatheter material during rotational atherectomy. Although no angiographic slow-flow, no-reflow, or immediate clinical sequelae were observed in this case, the downstream biological effects of such particulate exposure remain uncertain and warrant acknowledgment whenever this bailout strategy is considered.

This case highlights that, in selected complex calcified CTOs complicated by retained intraluminal fragments, procedural success may depend on reframing the objective from fragment retrieval to controlled plaque modification while preserving wire position and coronary flow.^1^^,^^6^

## Conclusion

In a severely calcified ostial LAD CTO, an entrapped microcatheter gold tip was managed with targeted plaque modification using rotational atherectomy, enabling completion of percutaneous coronary intervention with the final TIMI flow grade 3 (Figure 6).

**Take-Home Messages**

• Retention of a microcatheter distal tip is a rare but potentially catastrophic complication during percutaneous coronary intervention that requires immediate procedural reassessment.
• When direct retrieval is unsafe or unsuccessful, controlled plaque modification through the retained tip may restore procedural control provided a meticulous technique and surgical backup are available.

## Patient Consent

Written informed consent was obtained from the patient for publication of this case report, including accompanying images and videos.

## Funding Support and Author Disclosures

The authors have reported that they have no relationships relevant to the contents of this paper to disclose.

## Appendix

Video 1 Antegrade Wiring of the Left Anterior Descending Artery Chronic Total Occlusion After unsuccessful crossing with a Fielder FC guidewire, the lesion was successfully crossed with a Gaia Next II guidewire, with a second wire placed in the left circumflex artery for branch protection.

Video 2 Rotational Atherectomy with a 1.5-mm burr at 180,000 rpm Performed for Plaque Modification and Treatment of the Retained Microcatheter Distal Tip, With Progressive Fluoroscopic Reduction of the Retained Fragment
